# The need for multisectoral food chain approaches to reduce trans fat consumption in India

**DOI:** 10.1186/s12889-015-1988-7

**Published:** 2015-07-22

**Authors:** Shauna M Downs, Archna Singh, Vidhu Gupta, Karen Lock, Suparna Ghosh-Jerath

**Affiliations:** Menzies Centre for Health Policy, University of Sydney, Sydney, Australia; Indian Institute for Public Health, Public Health Foundation of India and All India Institute of Medical Sciences, Delhi, India; Indian Institute for Public Health, Public Health Foundation of India, Haryana, India; London School of Hygiene and Tropical Medicine and Leverhulme Centre for Integrative Research on Agriculture and Health, London, UK; Indian Institute for Public Health, Public Health Foundation of India, Plot No.34, Sector - 44, Institutional Area, Gurgaon, 122002 Haryana India

**Keywords:** Trans fat, Product reformulation, Trans fat policy, Multisectoral policy

## Abstract

**Background:**

The World Health Organization (WHO) recommends virtually eliminating trans fat from the global food supply. Although several high-income countries have successfully reduced trans fat levels in foods, low- and middle-income countries such as India face additional challenges to its removal from the food supply. This study provides a systems analysis of the Indian food chain to assess intervention options for reducing trans fat intake in low-income consumers.

**Methods:**

Data were collected at the manufacturer, retailer and consumer levels. Qualitative interviews were conducted with *vanaspati* manufacturers (*n* = 13) and local food vendors (*n* = 44). Laboratory analyses (*n* = 39) of street foods/snacks sold by the vendors were also conducted. Trans fat and snack intakes were also examined in low-income consumers in two rural villages (*n* = 260) and an urban slum (*n* = 261).

**Results:**

Manufacturers of *vanaspati* described reducing trans fat levels as feasible but identified challenges in using healthier oils. The fat content of sampled oils from street vendors contained high levels of saturated fat (24.7-69.3 % of total fat) and trans fat (0.1-29.9 % of total fat). Households were consuming snacks high in trans fat as part of daily diets (31 % village and 84.3 % of slum households) and 4 % of rural and 13 % of urban households exceeded WHO recommendations for trans fat intakes.

**Conclusions:**

A multisectoral food chain approach to reducing trans fat is needed in India and likely in other low- and middle-income countries worldwide. This will require investment in development of competitively priced bakery shortenings and economic incentives for manufacturing foods using healthier oils. Increased production of healthier oils will also be required alongside these investments, which will become increasingly important as more and more countries begin investing in palm oil production.

## Background

The consumption of industrially produced trans fat has been associated with an increased risk of cardiovascular disease [1–3]. Given that cardiovascular disease is the main contributor to the global burden of disease [4], reducing the availability of trans fat in the food supply is an important global public health priority.

Partially hydrogenated vegetable oils (PHVOs) are the main source of trans fat and are used as bakery shortening, as frying oil and, in some cases, in household cooking. Its use has been favoured by industry due to its long shelf life and low cost [5, 6]. However, the health consequences of its consumption are clear – a 2 % increase in energy intake from trans fat has been associated with a 23 % increase in the risk of heart disease [1]. This evidence has led the World Health Organization (WHO) to recommend limiting trans fat intakes to less than 1 % of total energy intake [7]. The Global Monitoring Framework for non-communicable disease (NCD) control and prevention proposes national policies that virtually eliminate PHVOs from the global food supply. The WHO recommends replacing PHVOs with unsaturated fat in order to achieve the greatest population health benefit [8]. However, some low- and middle-income countries (LMICs) may consider this unfeasible despite evidence to the contrary from high-income countries [9].

Over the past decade, India has experienced rapid economic growth, which has resulted in socioeconomic, demographic, nutrition and health transitions [10]. Along with the persistent high rates of childhood undernutrition, there has been a rapid rise in diet-related NCDs, affecting all sections of society including those from lower socio-economic groups [10, 11]. One of the reasons for the rapid rise in NCDs in India has been a rapid change in dietary patterns. In recent decades there has been an increase in consumption of edible oils, eggs and meat among both rural and urban Indians [12]. The consumption of edible oil has risen by 9.7 million tons in 2000–01 to 14.3 million tons during 2007–08 [13]. Of this oil, approximately 10 % is *vanaspati*, − a vegetable ghee PHVO which is high in trans fat – 35 % is raw oil and 55 % is refined [13, 14]. Between 1972–72 and 2004–05, fat consumption increased from 24 g/person/day to 36 g/person/day capita in rural areas and 36 g/person/day to 47.5 g/person/day in urban areas [13]. Concurrently with the rise in fat consumption, the consumption of processed foods (high in sugar and fat) and sugar sweetened beverages has risen in urban populations while the number of meals prepared and consumed at home has decreased as eating out becomes more popular [15]. More specifically, between 2009 and 2014 there was a 37 % increase in volume growth of packaged foods and a 21 % increase in volume growth of soft drinks [16, 17].

The removal of trans fat from the global food supply has been deemed one of the most straightforward public health interventions to reduce NCDs [16]. Trans fats are not essential dietary components. Reduction in trans fat intakes at the population level has been achieved in high-income countries through a combination of mandated trans fat labelling, public education campaigns, engagement with industry to reformulate products and regulation of levels of trans fats nationally and locally [17, 18], the latter being the most effective [9]. LMICs such as India are likely to face additional challenges to trans fat reduction related to the large informal food sector which includes the small manufacturing enterprises and small traders and service providers, legal and illegal activities and a wide array of artisans [21]. These include reduced capacity for enforcement, lack of coordination among policy sectors, competing health priorities and lack of awareness regarding trans fat [18–23]. Nevertheless there are examples of success in reducing trans fat in the food supply in Latin American countries such as Costa Rica and Argentina. Costa Rica adopted voluntary approaches to reducing trans fat in the food supply by actively engaging with the oil industry and Argentina worked closely with the agricultural industry to ensure that there was an adequate supply of healthier replacement oils for PHVOs [14, 20, 24, 25]. These approaches were successful. In Costa Rica, the amount of total trans fat in soybean oil (previously partially hydrogenated) decreased from an average of 20 % to 1.5 % [26] while in Argentina approximately 40 % of the 30, 000 metric tons of trans fat that were produced annually were replaced with other fats in a very short period of time [14].

In July 2013 the Government of India published trans fat regulation that would require manufacturers of PHVOs such as *vanaspati* to limit the trans fat content to 10 % and require trans fat labeling on packaged food. More recently, in December 2014, the Government of India published revised trans fat regulation that would require manufacturers to reduce trans fat levels to 5 % in PHVOs by August 27, 2016 [27].

The aim of the research was to provide a systems analysis of the Indian food chain to assess intervention options for reducing population trans fat intake. This paper provides an overview of the findings of a mixed methods study that examined three levels of the Indian food supply – the manufacturers, retailers and consumers. The specific study objectives were: at the manufacturing level to examine the feasibility of lowering trans fat content in PHVOs; at the retail level to describe the food environment of two low socio-economic status (SES) communities in a rural and urban slum setting in India with specific attention to trans fat and to analyze trans fat levels in the commonly consumed snacks from vendors in the communities; and at the consumer level to estimate the proportion of daily energy intake contributed by trans fat in an urban and rural low SES community.

## Methods

### Overview of methodology

This project adopted a novel methodological approach to examining reduction of trans fat intake within the Indian context. Mixed-methods including qualitative interviews with *vanaspati* manufacturers and local food vendors, quantitative methods to assess dietary intake and laboratory analyses of food were used to gain a detailed analysis of the opportunities and challenges for trans fat reduction at the manufacturer, retailer and consumer level of the Indian food supply. We provide an overview of the methods used below; a detailed description can be found elsewhere [28, 29]. Ethics approval for the study was obtained from the University of Sydney and the Public Health Foundation of India’s Institutional Ethics Committees.

### Manufacturing Level

Thirteen semi-structured interviews were conducted with respondents who had technical expertise on the use of PHVO in the Indian food industry. Industry interviewees were either the Technical or Research and Development Directors of the manufacturing companies. Study participants were recruited using purposive sampling to provide perspectives of companies of varying size (i.e., multinationals and small and medium enterprises). Of the 79 manufacturers contacted, we were unable to make contact with 30 (mainly due to incorrect contact information) and 15 no longer manufactured *vanaspati*. Of the remaining 34 companies, ten participated in the study; however, these companies represented a large proportion of the market share. The interviews gained information about the technical and economic feasibility of reducing trans fat in PHVOs and replacing it with healthier oils (i.e., unsaturated fats). Interviews were conducted in English, with the exception of one interview, which was completed in Hindi.

### Retailer Level

We examined the local food environments of two adjacent villages in the North Indian state of Haryana and an urban slum setting in Delhi. The settings were purposively chosen to examine the food environments of low SES populations in rural and urban settings. All food vendors working in each of the study communities were invited to participate in the study. A structured survey was conducted with local food vendors (villages *n* = 27 (68 % participation rate), slum *n* = 17 (59 % participation rate)) from the study communities to gain an in depth understanding into the feasibility of changing oil use to make trans fat free products, their awareness of trans fat (i.e., had heard of trans fat and whether they were aware of the associated adverse health effects) in the foods they produce and the acceptability of product reformulation. All interviews were conducted in Hindi and verbal responses were recorded by research staff.

In addition to the interviews with retailers, laboratory analyses of the snack products they sold, and were commonly consumed, were also conducted. The commonly eaten local snacks were identified in the household surveys and samples of these snacks were collected for laboratory analysis. A total of seventeen snack samples from the villages and 32 from the slums were examined using gas chromatography (GC) to assess the quality of the fat present in snacks by analysing the fatty acid profiles of the extracted fats according to AOAC protocols (AOAC 996.06). The basic processing for fat extraction was conducted at the South Asian Network for Chronic Disease in India while the gas chromatographic analysis was completed at the All India Institute of Medical Sciences laboratory. The fatty acid profile analysis was conducted after trans-esterification of the extracted fats to their respective esters, which were subsequently run on a GC equipped with a flame ionization (FID) detector (Nucon Series II, 5700/5765). Individual fatty acid esters from NuChek Prep Inc. USA, were used to characterize and identify individual fatty acids (with respect to saturated, mono-, poly- and trans- unsaturated fatty acids) in the oils extracted from the snacks and C11:0 (undecanoic methyl ester) was used as an internal standard. The limits of detection and quantitation were calculated using the GLC-607 mix. All analyses were done in duplicates.

### Consumer level

A sample size of 260 urban and rural households was calculated to enable us to detect a difference of 2.5 % of total fat from trans fat, with a precision of 0.02, an alpha of 5 % and a non-response rate of 10 %. Every third household in both the village and urban slum settings were approached to participate in the household survey. Given that survey participation required visits from the research staff on three consecutive days, the participation rate was lower than expected. Overall, 65 % of rural and 50 % of urban households agreed to participate. Two hundred and sixty households in the villages and 261 households in the urban slum were included in the dietary intake survey. A pre-tested interviewer administered questionnaire was used for the household survey, consisting of questions related to the socio-economic and demographic profiles of the households, daily food consumption and snacking patterns. In addition, a 24-hour dietary recall was conducted by trained research assistants on two consecutive days in all households. The dietary consumption data was collected at the household level and was used to compute the average food and nutrient intake of the household members expressed as per consumption unit (CU) per day. Diet Soft (version 1.2.0; 2008–2009; Department of Dietetics; AIIMS & Invincible IDEAS Co., India) software, which is based on the Nutritive Value of Indian foods database [30], was used to estimate nutrient intakes. In addition to examining dietary intakes, heights and weights were measured and used to calculate body mass index (BMI). A BMI of 23 kg/m^2^ and 27.5 kg/m^2^ were used to classify overweight and obesity, respectively, as per WHO recommendations for Asian populations [31].

All statistical analyses were conducted in SPSS (version 20, IBM SPSS Statistics). Statistical differences in continuous variables between rural and urban households were examined using a Mann–Whitney U test for abnormally distributed variables and a t-test for normally distributed variables. A p-value <0.05 was considered statistically significant.

## Results

**Fig. 1:**
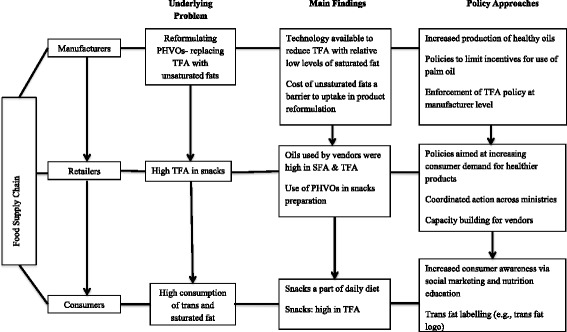
An overview of the main study findings and their policy implications

Figure 1 provides an overview of the study findings and their implications for policy. The specific results are reported below at the manufacturer, retailer and consumer level.

### Manufacturing level

Indian *vanaspati* manufacturers described reducing trans fat in *vanaspati* as being both technically and economically feasible; however, they indicated there would be challenges in meeting consumer preferences given that consumers demand products with specific organoleptic properties (i.e., consistency), particularly if manufacturers were to bring the trans fat levels down to 5 % of total fat. Smaller manufacturers described having less capacity to reformulate their products and less likely to adhere to regulation. Manufacturers indicated that they would reformulate their product using palm oil (which was already being used in high quantities), which will decrease the trans fat but increase the saturated fat levels. Reformulating *vanaspati* and bakery shortening using ‘healthier’ unsaturated fat was seen as a challenge for Indian industry due to increased costs associated with its use, and low availability within the country in contrast to abundance of cheaper palm oil supplies [28].

### Retailer level

Overall, trans fat awareness was low (n = 3, 7 %) among vendors. The majority of vendors were sourcing their snacks from wholesalers (without any knowledge of the source of fat used in the products) and many of the products did not contain nutrition information labels. Most urban vendors indicated that they would be willing to change the type of oil used provided the taste, cost and customer acceptability remained unchanged; rural vendors were more resistant to change. Of the rural and urban vendors who prepared snacks themselves, they used a variety of different oils including soybean and other non-specified refined oils, *vanaspati* and local oil brands. The fat content of sampled oils (n = 11) from the vendors ranged from 24.7-69.3 % of total fat from saturated fat (mean 51.6 % ± 16.5 % SD) whereas trans fat levels ranged between 0.1-29.9 % of total fat (mean 8.7 % ± 11.2 % SD). Trans fat levels exceeded 20 % of total fatty acids in both the urban and rural *vanaspati* samples (n = 2) and in the sample of *desi ghee* taken from the village market.

### Consumer level

The main oils used by households in both the rural and urban settings were similar: soybean and mustard oil, *vanaspati* and butter. In addition rural households used *desi ghee*. Fifteen percent of households in slums (n = 38) and 11 % households (n = 29) in the villages reported consuming *vanaspati,* along with other cooking mediums. Of those households that reported using *vanaspati* as cooking oil, the reported median consumption was 24 grams in urban and 7 grams in rural areas. The major source of dietary trans fat in the survey was from the consumption of commercially prepared snacks (i.e., consumed away from home). Thirty-one percent (n = 82) of households were consuming snacks containing high trans fat in villages as compared to 84.3 % (n = 220) in slums. The median total fat consumption was higher in the urban slum (36.9 g/CU/day) as compared to the rural villages (29.5 g/CU/day; p < 0.001) and a larger proportion of fat was attributed to snack consumption in the urban slum (median 10.8 g/CU/day, range 0–90.2 g/CU/day) than in the rural villages (median 1 g/CU/day, range 0–58.5 g/CU/day; p < 0.001). The median trans fat consumption was 0.22 g/CU/day (range: 0.01-14.79 g/CU/day) in the villages and 0.67 g/CU/day (range: 0.01-11.44 g/CU/day) in the slums (p = 0.001). The majority of the households did not exceed the WHO recommendation to limit trans fat intakes to less than 1 % of total energy; however, 4 % (n = 10) of village households and 13 % (n = 33) of urban households consumed more than 1 % of total energy from trans fat. The prevalence of overweight/obesity was higher in urban households where 67 % of adults were overweight or obese as compared to 28 % in rural households.

## Discussion

This study provides the first comprehensive food chain study of the trans fat policy context in India and provides insight into the most appropriate interventions to reduce population intakes. It provides a novel overview of the feasibility of reducing trans fat in PHVOs from the perspective of food manufacturers, insight into local food vendor practices and an estimate of trans fat use and consumption in low SES consumers in India.

### Trans fat availability in the food supply

Foods and oils containing high amounts of trans and saturated fat remain in the Indian food supply, which has previously been shown in studies in India [32, 33]. This is problematic given that we found that many poor households were frequently consuming these high trans fat snacks. There are no nationally representative samples that examine trans fat intakes in India; however, our study suggests that much like other countries worldwide, there are pockets of the populations – including those who are younger and more socioeconomically disadvantaged – who consume unhealthy levels of trans fat [34, 35]. Therefore a combination of policies that actively target manufacturers, including those in the informal sectors, provides all retailers including low-income food vendors with access to competitively priced oil that contain the organoleptic properties that consumers demand, and improves consumer and vendor awareness of the trans fat content and health impacts of snack foods will be needed.

### Policy at the manufacturer level

The most effective way to reduce trans fat availability in the food supply is to ban it. Although there have only been a small number of countries that have been able to enact a ban, this approach to reducing trans fat in the food supply has been successful [6, 9]. Alternatively, countries such as Canada and the United States have been able to significantly lower trans fat levels in the food supply with mandatory trans fat labeling resulting in product reformulation [9, 36–38]. However, the Indian context is very different to other countries that have successfully reduced the trans fat content of foods. Given the sheer scale and diversity of the country, lack of capacity for enforcement and the informal (and largely unregulated) food sector in India [19], a trans fat ban may not have the anticipated impact on trans fat levels in the Indian food supply. Although ensuring that such policy measures are adequately enforced – and corruption minimized – could improve their effectiveness, additional measures may be needed in order to reduce trans fat availability across the whole food supply.

Although *vanaspati* manufacturers in India have indicated that it is feasible to reduce trans fat in their products to 10 % they would accomplish this by relying more heavily on palm oil. Although this would likely be associated with improved health outcomes [39] additional health benefits may be possible with reformulation using oils high in unsaturated fats [1, 40]. They also indicated difficulties in bringing down levels to 5 %. The technology exists to produce products similar in consistency to *vanaspati* that are trans fat free and relatively low in saturated fat [41, 42]; however, currently widespread adoption has not taken place in India. Increasing investment into development and mass production of cost-effective bakery shortenings and frying oils that have a healthier fatty acid profile and are affordable could be a key way to reduce the use of PHVOs and increase uptake of healthier oils. However, investment in technology will need to be coupled with investment in agricultural supply chains of healthier oils in order to allow manufacturers to replace PHVOs with oils high in unsaturated rather than saturated fat (i.e. alternatives to palm oil). In the 1970s, Brazil increased soybean production substantially by investing public funding for soybean breeding, minimum price supports, production and marketing credit programs, agricultural subsidies, public infrastructure programs and supportive energy and taxation policies [43]. This resulted in soaring production leading to a shift in fat consumption from animal fats to soybean oil [44]. Given that India relies heavily on imports of palm oil due to the low productivity of healthier oils produced domestically, improving inputs into Indian agricultural production is a key policy intervention if trans fat reduction is to succeed [45].

### Policy at the retailer level

We found that many of the vendors in the communities studied were not producing the food themselves but were instead purchasing snacks from wholesalers. Vendors who prepared snacks themselves used various different types of oils, many of which were high in either saturated, trans fat or both. One of the challenges faced by retailers is that the oils they purchase often do not contain nutrition labels making it difficult, if not impossible, for them to make informed purchasing decisions based on the quality of the fat. Although the majority of vendors were not aware of trans fat, even if their awareness increased, they would not be able to choose healthier oils without increased transparency in labeling. This is further compounded by the fact that it appeared that some oils might have been adulterated. For example, the *desi ghee* sample from the village market had trans fat levels that resembled *vanaspati* rather than ghee. It is clear from these findings that more needs to be done at the manufacturer level to ensure that oils are labeled correctly.

In order to ensure that both retailers and wholesalers prioritize the quality of the oil used, there is a need to stimulate greater consumer demand for products using healthier oils while potentially concurrently incentivizing manufacturers, wholesalers and retailers to offer new products at comparable costs. This requires coordinated policy action from Public Health, Economic and Business ministries. For example, in Singapore the Health Promotion Board (HPB) began an initiative in 2011 called the Healthier Hawker Program, which aims to reduce the saturated fat content of cooking oils used by food vendors [46]. In order to ensure that there was an affordable supply of healthier oils, the HPB worked with local manufacturing companies to increase the supply of blended oils containing 25 % less saturated fat [46]. In order to cut costs for vendors, they established cooperatives where manufacturers sold and vendors bought these healthier oils. By streamlining this supply chain, it reduced the price, making it a competitive option for vendors. In order to highlight those vendors using the healthier oil, they also adopted a healthier ingredients symbol program – which is part of the program – allows vendors to put up a sign to indicate use of healthier ingredients if trans fat levels are less than 0.5 g/100 g and saturated fat levels are less than 38 g/100 g [46, 47]. A similar initiative could take place in India, particularly in urban areas, to try to increase access to healthier oils by local food vendors. This type of intervention would likely need to take place at the municipal level, which may increase the likelihood of policy uptake. Importantly, the funding for the Singapore Healthy Hawkers program did not come out of the health budget but rather from an economic initiative to support small and medium enterprises [46, 47], pointing to the need to be innovative in terms of identifying opportunities to support multisectoral policy approaches.

### Policy at the consumer level

In addition to intervening further upstream in the Indian fats supply chain, interventions aimed at increasing consumer awareness are needed. In the USA and Canada, mandatory trans fat labelling was associated with a significant reduction in the availability of trans fat in the food supply [19, 48–51]. This was aided by increased consumer awareness regarding trans fat. Increased consumer education and trans fat labelling in India is needed alongside the more upstream policy approaches particularly given that products containing trans fat do not always include a nutrition label (i.e., street food). A simple labelling system (such as a trans fat logo) could help increase consumer awareness related to trans fat, thereby increasing consumer demand for use of healthier oils by both manufacturers and vendors.

### Limitations

Although there are many strengths to the multilevel, mixed methods approach used in this study there are also important limitations including the small sample sizes, a limited geographical focus and reliance on household rather than individual level dietary intakes. The small sample size and limited geographical focus have important implications for the generalizability of the study findings. It is likely that trans fat intakes are quite variable across India – additional research examining trans fat intakes, as well as its dietary sources, is required on a larger scale. However, this study provides insight into the main sources of *vanaspati* consumption and the strategies that would be most effective in addressing its production, sale and consumption in low-income populations in rural Haryana and an urban slum in Delhi.

Lastly, there are inherent limitations to conducting 24-hour dietary recalls including their inability to account for day-to-day variation in intakes [52]. Although we tried to minimize this limitation by conducting dietary recalls on two consecutive days it could have led to reported trans fat intakes that differed from usual intakes.

## Conclusions

The Government of India has published regulations to limit trans fat in the food supply, but it needs a proactive approach to ensure that the regulation will have the intended effect. Low SES populations in India are consuming snacks high in trans fat and some are exceeding international dietary recommendations. Given the size and diversity of India, and its informal manufacturing and retail sectors, national bans, voluntary industry agreements or nutrition labeling that have been effective in other countries are unlikely to be successful if used in isolation [9]. India requires a multisectoral policy approach with coordinated action at the agricultural production, manufacturing, retail and consumer levels. It is likely that other low- and middle-income countries will require a broader approach such as the one required in India. Ensuring that Ministries and regulatory bodies work together in a cohesive and concerted way to address the quality of the food supply will be essential. Increased investment in research and development to produce competitively priced bakery shortenings and frying oils that are trans fat free and relatively low in saturated fat is needed alongside investment in agricultural production of healthier oils. Manufacturers and food vendors need economic incentives for producing products using healthier oils, while improving consumer awareness of health issues through labeling will likely lead to changing consumer demand. Policies that aim to improve the quality of fat consumed also need to take place within the broader context of improving diet quality at the population level by tackling the double burden of diet-related disease. This is particularly important given the high and increasing rates of overweight and obesity in low-income populations in India and elsewhere worldwide.

## Abbreviations

WHO: World Health Organization
PHVO: partially hydrogenated vegetable oil
NCD: non-communicable disease
LMIC: low- and middle-income country
SES: socioeconomic status
GC: gas chromatography
CU: consumption unit
BMI: body mass index
HPB: Health Promotion Board
